# A cognitively enhanced online Tai Ji Quan training intervention for community-dwelling older adults with mild cognitive impairment: A feasibility trial

**DOI:** 10.1186/s12877-021-02747-0

**Published:** 2022-01-25

**Authors:** Fuzhong Li, Peter Harmer, Kathleen Fitzgerald, Kerri Winters-Stone

**Affiliations:** 1 Oregon Research Institute, Eugene, Oregon, OR 97403 USA; 2 Department of Exercise and Health Science, Willamette University, Salem, OR 97301 USA; 3 McKenzie Willamette Medical Center, Springfield, OR 97477 USA; 4 Knight Cancer Institute, Oregon Health & Science University, Portland, OR 97239 USA

**Keywords:** Cognitive impairment, Dual-task, Elderly, Exercise, Tai Chi, Telehealth

## Abstract

**Background:**

This study examines the feasibility, acceptability, and safety of a newly developed cognitive-enhancing Tai Ji Quan training intervention, delivered via remote videoconferencing, for older adults with mild cognitive impairment (MCI).

**Methods:**

In a three-arm feasibility trial, community-dwelling older adults with MCI (N = 69; mean age = 74.6 years, 57% women) were randomized to a cognitively enhanced Tai Ji Quan (n = 23), standard Tai Ji Quan (n = 22), or stretching group (n = 24) and participated in a 60-minute online exercise session via Zoom, twice weekly for 16 weeks. Participants were recruited primarily in the state of Oregon through mass mailing and word of mouth. The primary outcomes were intervention feasibility (with respect to recruitment, online intervention delivery, fidelity and compliance, and attrition and retention rates), acceptability, and safety. We also assessed feasibility of online data collection and test-retest reliability and explored preliminary trends on secondary outcomes that included global cognitive function, dual-task cost, and domain-specific cognition function.

**Results:**

The study had an average recruitment rate of 55%. Feasibility was demonstrated by the overall successful online program implementation, with good fidelity, acceptable compliance (76%), and excellent retention (94%). The cognitively enhanced Tai Ji Quan intervention was shown to be acceptable to participants as well as safe, with no major intervention-related moderate/severe events. At week 16, the group receiving cognitively enhanced Tai Ji Quan training showed a positive trend in the cognitive function and dual-task outcome measures whereas the group receiving standard Tai Ji Quan training exhibited positive trends on global and domain-specific cognitive measures.

**Conclusions:**

Preliminary findings of this pilot study indicate the feasibility, acceptability, and safety of a tailored, cognitively enhanced Tai Ji Quan training intervention delivered remotely to home settings via videoconferencing for community-dwelling older adults with MCI.

**Trial registration:**

Clinicaltrials.gov identifier NCT04070703

## Background

Age-related decline in cognitive function has long been recognized as an important part of the aging process [1]. Early symptoms of cognitive decline include memory loss [2] that may lead to a condition commonly known as mild cognitive impairment (MCI) [3]. Progressive deterioration of MCI will ultimately put aging adults, whose health and quality of life are often compromised by acute or chronic health conditions, at high risk for developing or progressing to Alzheimer’s disease or a related dementia [3, 4]. Successfully addressing this global public health crisis [5, 6], however, requires intervention strategies that can effectively prevent or delay any decline in cognitive function, thus decreasing dementia risk [7].

Non-pharmacological interventions, such as physical exercise, have been shown to improve cognitive performance, including memory, processing speed, and executive function in older adults with and without cognitive impairment [8–10]. Research suggests that the cognitive benefits may be further enhanced through integration of concurrent cognitive and motor task activities, that is, dual-task training [11–13]. In this respect, Tai Ji Quan, which requires a significant amount of cognitive effort allocated to the performance and mastery of intricate whole-body movements [14, 15], appears to fit well as a cognitive and motor training therapy for preventing or mitigating memory loss and cognitive decline among older adults with MCI [16–18].

Contemporary Tai Ji Quan training approaches, which primarily emphasize training participants to master Tai Ji Quan forms and movements [14], do not explicitly integrate the cognitively demanding features of this mind-body therapy into a multitasking exercise that could be cognitively stimulating and mentally beneficial for the MCI population, which experiences increasing difficulties in performing dual-task functions in activities of daily living [19, 20]. In addition, existing Tai Ji Quan intervention delivery methods are primarily group-based with in-person administration. This approach can create barriers for practical implementation and broad dissemination, especially in extenuating circumstances, such as the COVID-19 pandemic, which has significantly restricted daily life activities of older adults, including attending community-based exercise classes [21].

In an effort to integrate cognitive activities explicitly into Tai Ji Quan training in order to maximize its cognitive training benefits, we developed a cognitively enhanced Tai Ji Quan intervention, delivered online via Zoom videoconferencing, for community-dwelling older adults with MCI. In this pilot study, our objectives were to (a) examine the feasibility (with respect to recruitment, online intervention delivery, fidelity, compliance, and attrition and retention rates), acceptability, and intervention safety of the newly developed intervention, (b) assess the feasibility of online data collection and test-retest reliability, and (c) explore preliminary trends in cognitive function and dual-task cost data to inform future full-scale trials.

## Methods

### Study Design

We performed a single-blind, 3-arm, parallel design, randomized feasibility trial with a 1:1:1 allocation ratio. The feasibility study was conducted in conjunction with an on-going trial (Clinicaltrials.gov identifier NCT04070703; 28/08/2019), with the research protocol approved by the institutional review board of the Oregon Research Institute. The study was performed between August 2020 and September 2021, during the 2020-2021 phase of the COVID-19 pandemic, with all research activities (recruitment, enrollment, intervention, and data collection/assessment) performed remotely via the use of telephones (for prescreening) and online conferencing software (Zoom.com) (for trial implementation and outcome assessment). All participants provided informed consent, completed electronically via online Qualtrics survey software (qualtrics.com). The feasibility-trial reporting follows the general guidelines described in the Consolidated Standards of Reporting Trials (CONSORT) 2010 statement: Extension to randomized pilot and feasibility trials [22].

### Setting, Participants, Recruitment, and Procedures

The study was undertaken in home settings where the target population was community-dwelling, independent-living older adults. Initial enrollment efforts targeted individuals who lived in Oregon but was expanded to other states in the U.S. after the first wave of recruitment. Multiple methods of recruitment were used, including direct mailing, online promotions (e.g., research website, social media), word of mouth, and contact with volunteers from previous research projects.

The recruitment criterion was community-dwelling adults aged 65 years or older who met diagnostic criteria for having MCI [23]. Evidence of MCI was established if individuals (a) self-reported a change or decline in memory (corroborated by an informant), (b) recorded a score of ≤0.5 on the Clinical Dementia Rating (CDR) [24] scale (with information derived from both the participant and an informant), and (c) showed no major impairment in cognitive function as indexed by a score of ≥24 on the Mini Mental State Evaluation (MMSE) [25]. We excluded individuals who (a) had participated in a rigorous and structured physical activity or exercise program (including Tai Ji Quan) in the past 3 months, (b) showed signs of depression as indicated by a score of ≥5 on the Geriatric Depression Scale [26], (c) had major medical or physical conditions that precluded exercise, as determined by their healthcare practitioner, or (d) were unable to follow the consent process or sign the study consent form.

Recruitment was conducted primarily via initial phone communication, followed by a scheduled online Zoom meeting using a procedure established in a prior study [27]. Briefly, a research assistant made initial contact via a telephone call with those who responded to the study promotion. This contact confirmed eligibility criteria regarding age, memory status, levels of habitual physical activity, and time availability. An independent interview was conducted with an informant who provided information regarding the respondent’s cognitive status based in part on a CDR assessment [24]. Individuals were further evaluated, via an online Zoom session, on additional eligibility criteria, including administration of the CDR and MMSE. During each stage of prescreening, individuals were given sufficient time to ask questions and clarify issues related to risk, benefits, and commitment related to study participation. Those who met the study eligibility criteria signed the study consent form, and then underwent an online baseline assessment session conducted by an independent assessor. The recruitment for the feasibility study ended, as planned, in April 2021.

### Randomization and Blinding

Eligible individuals were randomly assigned in a 1:1:1 ratio to receive one of the three active interventions, with a block size of 4 or 6. Randomization was not stratified. The randomization list was generated by a research data analyst with the use of nQuery software (www.statsols.com) and was kept in sealed envelopes. Allocation concealment was ensured since the randomization was performed by a research assistant who did not know the sequence of interventions and was not involved in the assessment or intervention. Randomization occurred after informed consent was obtained and baseline assessments were completed.

Participants were not blinded to the intervention group allocation but were requested not to disclose their group allocation to the study assessors during all follow-up assessments. Outcome assessors were masked to group allocation throughout the study period. Blinding was strictly maintained by (a) emphasizing to assessors the importance of minimizing assessment bias, (b) regular checking of the blinding status via inquiries made to randomly selected participants, and (c) randomly asking assessors to guess what intervention the participants had received. Every effort was made to keep the assessors from knowing the group allocation, including the use of identical assessment forms, assigned study participation identification numbers, and a designated database separate from the one used by unblinded study recruiters. In instances when blinding was broken, the assessor was immediately excused and replaced by another blind assessor.

### Intervention

#### Cognitively Enhanced Tai Ji Quan Training

The intervention was adapted from the Tai Ji Quan: Moving for Better Balance program [14, 28], augmented by a set of cognitively stimulating exercises practiced concomitantly with Tai Ji Quan to increase cognitive processing demands [27, 29]. The progressive training consisted of 5-15 minutes of preparatory (warm-up) exercises, 45-55 minutes of core training (learning, practicing, repeating) in the 8-form routine [28], and 1-2 minutes of cool-down exercise. During the initial 10 weeks, the core physical exercise involved primarily learning and repeatedly practicing the 8 forms (in a prespecified sequence of *Moving a Ball*, *Part Wild Horse’s Mane*, *Repulse Monkey*, *Brush Knees*, *Fair Lady Works the Shuttle*, *Grasp Peacock’s Tail*, *Waving Hands like Clouds*, and *Single Whip*), with training focusing on symmetrical and coordinated actions such as trunk rotation and weight shifting, controlled and coordinated displacement of the body’s center of mass over the base of support, dynamic eyes-head movements, and anterior-posterior and medial-lateral stepping. From week 10 on, training became fully integrated with prespecified cognitive exercises, described below, that targeted domains of memory, executive function, and processing speed.

The training approach used a mix of dual-task, task prioritization, and memory exercises intended to actively engage participants in learning and practicing Tai Ji Quan forms and variations of forms and encouraging instructor-participant interaction during practice. Specifics of integrated Tai Ji Quan exercises included (a) verbalizing step-by-step movements involved in a form, (b) responding to correct form instructions while inhibiting unwanted instructions, (c) exercising form recalls (including 2 or 3 forms back) either standing (with a narrow base of support) or moving, (d) practicing a sequence of forms while verbally associating form names with form numbers (or the reverse), (e) performing forms without verbal or visual instructional cues, and (f) performing word spelling (forward and backward), side-switching (alternating between lateral and bilateral sides), out-of-sequence form switching, and varying form sequences (forward, backward, oddly or evenly ordered).

#### Standard Tai Ji Quan Training

This intervention consisted of the identical 8-form routine training protocol [28] implemented for the cognitively enhanced Tai Ji Quan group, except that the cognitive exercises were not included. Instead, an integrated set of therapeutic movement exercises was added that focused on training of sensory integration, mobility, and limits of stability and recovery of near loss of balance. These exercises followed the general guidelines regarding balance training for older adults, with activities that emphasized progressively difficult postures with varying sizes of the base of support, postural perturbation that displaced the body away from equilibrium, weight-loading on lower-extremity postural muscle groups, and sensory compensation and integration [30].

The intervention followed a traditional Tai Ji Quan training approach, where instructors play a dominant and leading role in teaching and practicing, without instructor-participant interaction during exercise. Accordingly, each exercise session primarily involved teaching participants, via both verbal and visual cues, Tai Ji Quan forms and movements through repeated practice. The specifics of physical training activities included controlled, self-initiated Tai Ji Quan–based exercises with synchronized breathing, including center-of-gravity displacement using a dynamic interplay of stabilizing and self-induced destabilizing postural actions involving unilateral weight-bearing and weight-shifting movements, trunk and pelvic rotation, ankle sway, multidirectional stepping, and eye-head-hand movements [28]. Similar to the protocol of the cognitively enhanced group, the exercise training in this intervention was taught progressively, with learning and practice of the 8 individual forms taking place during the first 10 weeks and repeated practice with variations thereafter.

#### Stretching Exercise

This intervention, with the protocol adopted from our prior trial [28] and piloted in another study [27], consisted of various muscle stretches, performed in either seated or standing positions. The teaching strategy was identical to that of standard Tai Ji Quan training in that participants were asked to follow instructors’ instructions in performing all exercise activities specified in the program, and no instructor-participant interaction was encouraged. Each session began with light warm-up exercises involving arm, neck, and leg circles; trunk rotation; and light walking. The core part of the exercises involved stretches of the upper body (neck, arms, back, shoulders, and chest) and lower extremities (quadriceps, hamstrings, calves, and hips), along with slow and gentle trunk rotations. Each exercise session ended with breathing exercises and progressive relaxation of major muscle groups.

### Online Intervention Delivery Protocol

The three active exercise interventions were delivered using Zoom, for 16 weeks, with the same length of time (60 minutes per class session) and frequency (twice weekly). On each class day, participants were given a secured Zoom link via e-mail that allowed them to access the scheduled online exercise session. Participants were encouraged to sign into the online class 15 minutes before it started and, as a human subjects protection feature, they were admitted to the class by either the class instructor or project staff. After signing in, participants were instructed to (a) turn on video and (b) use the Speaker View feature in Zoom for better viewability of exercise instruction. During the class, participants in the standard Tai Ji Quan and stretching classes were encouraged to mute their audio for better audio quality. This was not done for those in the cognitively enhanced Tai Ji Quan group because of the need for interaction between the instructor and participants.

Exercise instructors trained by the first author taught all exercise sessions. All instructors were recruited from the community and had at least 10 years of teaching exercise or Tai Ji Quan experience. For consistency, in all three interventions, each session consisted of warm-up exercises, core exercises, and a brief cool-down activity. Exercise intensity in each intervention group was monitored through a subjective measure of perceived exertion, ranging from light to moderate for both Tai Ji Quan groups and light for the stretching group.

We began the study with a maximum of 8 participants per class to optimize viewability in the Zoom environment. We gradually increased the maximum number of participants in a class to 12-15 in subsequent waves of classes. We set the instructor’s teaching space to an area approximately 4 feet by 4 feet square in order to provide (a) onscreen viewability, (b) instructional clarity, and (c) effective space utilization and movement safety. On the participant end, we set an optimum physical distance of 8–10 feet for personal computer users and 6–8 feet for iPad or smart phone users for better viewability. Participants in each group were asked to have an armless chair set aside for use during exercise.

### Measurements

#### Primary Outcomes

***Intervention fidelity***. The intervention fidelity, as part of the quality control over intervention delivery, was defined as the extent to which interventionists successfully implemented the prespecified teaching protocol [31]. Our fidelity evaluation included (a) successful completion of the 60-minute session delivered twice weekly over a 16-week period, (b) the instructor’s adherence to the teaching and training protocol described in detail in our predeveloped teaching manual, and (c) an overall class attendance rate of ≥75% (out of the total planned 32 intervention class sessions). The items in a and b were monitored weekly by means of observing class attendance and conducting a weekly online class fidelity check. Items in a and b were also monitored by means of class recordings, which included an evaluation of the instructor’s faithfulness in implementing the key components of the interventions, involving warm-up, core exercise training, cool-down, and clarity in verbal and visual instructions. The fidelity check outcomes were as follows: (a) Pass Satisfactorily (faithful implementation), (b) Needs Attention (signs of deviations or drift and, therefore, adjustments needed), or (c) No Pass (in-service training needed) (see below).

Monthly in-service training sessions conducted by the first author were planned to assure the quality of delivery. The training involved reviewing sessions taught in the previous weeks, correcting observed interventionist drift from the prespecified teaching activities/forms, reinforcing appropriate teaching techniques and steps (i.e., demonstration, learning/practice, repetitions, emphasis of key training points), doses (exercise sets, repetitions), and quality (visual and verbal instructions and presentation). The sessions also included a brief discussion of any safety issues related to the teaching and practicing of movements in the teaching manual.

***Intervention attrition and study retention*** **.** On the basis of our prior trials [27, 28, 32], we set a satisfactory a priori intervention attrition rate (i.e., participants who dropped out of the assigned intervention class before the 16-week termination) at ≤15%. A drop from the exercise class (intervention dropout) was defined as declining to continue participating in the class exercises either by notifying project staff (via e-mail or telephone) or not responding to any class participation reminders sent by project staff. We aimed at a 90% rate of retaining and assessing randomized participants with valid outcome data (i.e., with an expected ≤10% missing or incomplete on cognitive and dual-task gait performance outcomes) during the 16-week study period. The same definition for intervention dropout was applied to determine the study dropout rate.

***Acceptability*** **.** To assess the exercise program acceptability for the newly developed cognitively enhanced online Tai Ji Quan intervention, participants assigned to this group were asked to complete a 5-item attitudinal scale upon completing the class. The survey asked participants to rate their opinions about the class in the areas of exercise safety, challenge/enjoyment, appropriateness of exercise intensity, helpfulness in improving brain health, and overall program satisfaction. For the acceptability evaluation by the participants assigned to our cognitively enhanced Tai Ji Quan intervention group, we specified an a priori overall satisfaction level of ≥70%.

***Adverse events monitoring*** **.** Throughout the study period, both intervention- and non-intervention-related adverse events were closely monitored and recorded by research staff and adjudicated by the principal investigator of the study. We classified adverse events in three categories: Mild (i.e., events that required no medical treatment or were non-life threatening), Moderate (i.e., events that required medical treatment but were not immediate life-threatening conditions, and Serious (i.e., events that resulted in death or were life threatening and required medical treatment, including hospitalization, or significant disability/incapacity). For all events observed or reported, we further classified them into three categories in relation to the intervention: Unrelated (an event that was reported but not directly related to participation in the intervention), Possible (an event that was observed during a class that was considered likely to be associated with participation), or Definite (an event that was observed or reported during a class and was considered directly related to participation).

### Data Collection of Secondary Study Outcomes

We included a set of cognitive outcome measures ranging from global cognitive function and dual-task gait performance to several domain-specific cognitive measures. All outcome measures, which are described below, were collected remotely via Zoom.

The Montreal Cognitive Assessment (MoCA) [33] measures global cognitive function that encompasses multiple domains (attention, executive functions, memory, language, conceptual thinking, calculations, visuospatial abilities, and orientation). MoCA scores range from 0 to 30, with higher scores representing better cognitive functioning. The final MoCA scores were adjusted for level of education (i.e., 1 point was added to the total score of individuals with ≤12 years of education) [33].

The Timed Up and Go (TUG) test [34] was used to assess gait under single-task and dual-task conditions [35]. During the single-task condition, participants completed a 20-foot walk that involved (a) standing up from a chair, (b) walking (10 feet forward) at normal pace to a line on the floor, (c) turning, (d) walking back (10 feet) to the chair, and (e) sitting down. The 20-foot walk was then repeated under a dual-task condition where participants were asked to walk while performing an arithmetic task (i.e., starting at the number 81 and sequentially subtracting 5 from the resulting number). No specific verbal instructions were given for prioritization of one of the walking tasks during the dual-task walking trial. Using the average score (in seconds) of each walk, a dual-task cost measure was estimated through the following methods [35]:

Dual-task cost_baseline_ = (dual-task walking – single-task walking) / single-task walking x 100

Dual-task cost_16_weeks_ = (dual-task walking – single-task walking) / single-task walking x 100

Change in dual-task cost = dual-task cost_16_weeks_ – dual-task cost_baseline_

Change (measured in percentage) in dual-task cost from baseline was used to define dual-task cost at week 16, with positive values indicating deteriorated dual-task performance (i.e., dual-task cost), and negative values representing an improvement in dual-task performance with respect to single-task (i.e., dual-task benefit) [35].

The four domain-specific cognitive function measures were Trail Making Test B (TMT-B) [36] (in seconds), Category Fluency for Animals [37] (executive function), Forward Digit Span [38] (attention), and Backward Digit Span [38] (working memory). In TMT-B, participants were asked to connect numbers and letters in an alternating progressive sequence (i.e., 1-A-2-B-3-C, and so on) and scoring was expressed in terms of the time (in seconds) to completion. For Category Fluency, participants were asked to generate the names of as many animals as possible in 60 seconds. In assessing Forward and Backward Digit Span, participants were verbally presented with a series of digits (e.g., 6, 2, 9, 7) at a rate of one digit per second and were asked to repeat them verbatim. If participants succeeded, they were given a longer list (e.g., 5, 3, 8, 1, 6). The number of digits increased by one until the participant consecutively failed two trials of the same digit span length. In the Forward Digit-Span, participants were asked to repeat the digits in the given called order whereas in the Backward Digit-Span participants were asked to repeat the digits in reverse. The maximum number of points for each of these tests was 16 [38].

### Modifications and Test Consistency

To accommodate online assessment, modifications were made in some of the cognitive measures. Specifically, for the MoCA measure [33], in the Visuospatial/Executive section, participants were asked to verbally connect each letter to the corresponding number for the Trail Making task and draw both the object (e.g., cube) and clock on a piece of paper and, once completed, show them to the assessor for evaluation. In completing the “Read list of letters” task in the Attention section, the letter-tapping task was replaced by asking participants to count the number of A’s in the list. For the TMT-B measure [36], the hand-drawing task was replaced by asking participants to verbally alternate the letters and numbers out loud, and the assessor recorded the time to complete the task.

To assess test-retest reliability, we assessed the cognitive measures twice, using a 2-week interval. Test-retest reliability of the TUG measure was reported previously [27].

### Other Measures

In addition, we conducted a study health survey that contained participants’ demographic information, health or medical conditions, and levels of leisure physical activity [39] and depression [26]. The survey was collected via either online Qualtrics (qualtrics.com) or regular mail. Other information included self-reported weight, height, and blood pressure, which were obtained during an online assessment.

### Descriptive Data Analysis

Participant characteristics at baseline were summarized by intervention group using mean, standard deviation, or percentage. Intervention adherence was calculated as the percentage of preplanned intervention sessions (32 total) attended by participants. Responses on acceptability were tabulated to describe participants’ ratings of intervention acceptability in the cognitively enhanced Tai Ji Quan group. Similarly, observational or reported counts of any adverse events were tabulated by severity categories and their relationship to intervention. As a feasibility study, no inferential statistical analyses were performed [40]. As a result, we planned a sample of 60 (20 in each group) on the basis of our prior similar work [27].

For descriptive purposes, baseline and 16-week data related to cognitive and dual-task outcomes are described for each intervention group, with means and standard deviations. Trends data (i.e., differences between baseline and 16-week data) are presented as change scores, along with 95% confidence intervals. The intent-to-treat principle as used with all available data presented at baseline and 16 weeks. We performed a test-retest reliability analysis using the intraclass correlation coefficient (ICC) values. All data were processed or analyzed, where appropriate, using SPSS version 20 (SPSS, Inc.).

## Results

**Fig. 1:**
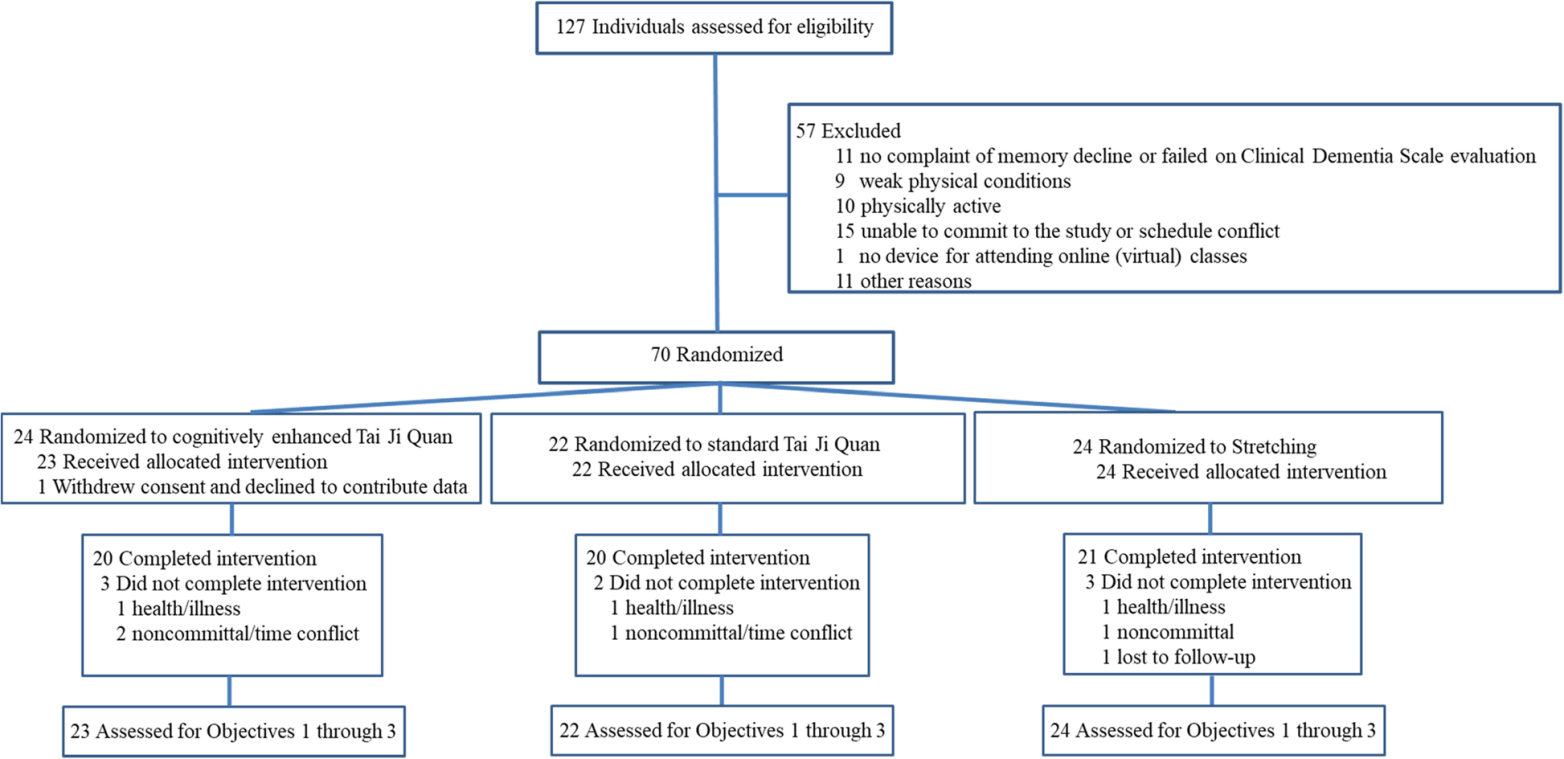
Flow of feasibility trial recruitment, enrollment, and randomization

Between August 2020 and April 2021, a total of 127 individuals were screened for eligibility; 70 were found eligible (a total recruitment rate of 55% [70/127]) and were subsequently randomized to one of the three intervention groups (Fig. 1). As part of our prescreening protocol for memory and cognitive-related impairment, we were also able to recruit the informant for these 70 participants. One participant was removed after withdrawing consent from the study, leaving a total of 69 participants who received an intervention. Baseline characteristics of the randomized participants by intervention groups are described in Table 1.

**Table 1:** Demographic characteristics of study participants by intervention group at baseline

Characteristic	Cognitively Enhanced Tai Ji Quan (n = 23)	Standard Tai Ji Quan (n = 22)	Stretching (n = 24)
Age – yr., mean (SD)	74.4 (5.1)	74.5 (5.6)	74.9 (6.3)
Female sex, No. (%)	16 (69.6)	8 (36.4)	15 (62.5)
Race/Ethnicity,^a^ No. (%)			
Non-Hispanic white	22 (95.7)	21 (95.5)	21 (87.5)
African American	1 (4.3)	0	0
Asian	0	0	1 (4.2)
Hispanic	0	0	1 (4.2)
Other	0	1 (4.5)	1 (4.2)
Education, No. (%)			
High school diploma or lower	12 (52.2)	10 (45.5)	13 (54.2)
College degree or higher	11 (47.8)	12 (54.5)	11 (45.8)
Body mass index,^b^ mean (SD)	29.9 (6.10)	27.7 (5.05)	27.9 (5.77)
Resting blood pressure - mm Hg, mean (SD)			
Systolic	125.65 (13.71)	126.73 (9.09)	125.92 (14.33)
Diastolic	73.78 (6.42)	72.91 (6.74)	72.42 (8.68)
Depression,^c^ mean (SD)	3.09 (1.31)	3.23 (1.66)	3.33 (1.24)
MMSE, mean (SD)	26.96 (1.87)	26.95 (1.91)	26.88 (2.19)
Self-reported chronic conditions, No. (%)			
None	2 (8.7)	1 (4.5)	1 (4.2)
1	5 (21.7)	10 (45.5)	5 (20.8)
2	5 (21.7)	4 (18.2)	5 (20.8)
≥3	11 (47.8)	7 (31.8)	13 (54.2)
Self-reported medication use, No. (%)			
None	7 (30.4)	7 (31.8)	7 (29.2)
1 medication	9 (39.1)	7 (31.8)	8 (33.3)
2 medications	5 (21.7)	6 (27.3)	4 (16.7)
≥3 medications	2 (8.8)	2 (9.1)	5 (20.8)

^a^Participants could respond “Yes” to more than one race/ethnic group.

^b^Calculated as the weight in kilograms divided by the square of the height in meters. SD: standard deviation.

^c^Geriatric Depression Scale (short form) consisting of 15 items (range: 0 to 15); 0-4 is considered normal, depending on age, education, and complaints; 5-8 indicates mild depression; 9-11 indicates moderate depression; and 12-15 indicates severe depression

The mean age for the study sample was 74.6 years (SD = 5.6; range = 65-92 years old), 57% were women, with 1.4% of participants being of Hispanic origin and 93% white. A total of 45 (65%) of the participants reported having 2 or more chronic conditions (e.g., arthritis, heart disease, high blood pressure, osteoporosis), and 24 (35%) participants reported taking 2 or more prescription medications. The sample had a mean (SD) MoCA score of 25.09 (2.47) and a mean (SD) MMSE score of 26.93 (1.97), with a relatively slow dual-task TUG performance (16.72 seconds [3.53]).

### Online Intervention Delivery

The 16-week online delivery of all three exercise intervention classes was successful with respect to the quality of exercise instructions by intervention instructors and their ability to implement the manualized online teaching protocols in an online environment. We also did not observe major interruptions or obstacles with respect to (a) receiving Zoom links from the project, signing on to Zoom, and operating Zoom (e.g., audio, video, viewability), (b) visibility and clarity in seeing the instructor’s teaching of exercise movements, or (c) availability of viewing devices for the class (i.e., personal computer, iPad, or Smart phones). While all study participants had Internet access and were able to attend classes, issues related to Internet connectivity occurred irregularly, a situation mostly impacted by either speed or weather conditions. Only one participant borrowed an iPad from us for class use. Because of the online delivery, the protocol allowed participation by those (n = 7) who were traveling or were away from their main homes during the course of the study.

### Intervention Fidelity and Compliance

Intervention fidelity was demonstrated by the fact that (a) all instructors successfully delivered the twice weekly, 16-week interventions and (b) there was an average compliance (class participation) rate of ≥75%. The average intervention attendance rate across the 16-week period for all intervention participants was 75.8% (76.6% in the cognitively enhanced Tai Ji Quan group, 75.5% in the standard Tai Ji Quan group, and 75.3% in the stretching exercise group). This rate met our a priori expectations. In addition, our weekly fidelity check on implementing intervention activities showed that all instructors passed the fidelity check satisfactorily and were able to follow and deliver the assigned intervention consistently and reliably per the activities detailed in the teaching plan.

### Intervention Attrition and Study Retention

A total of 8 participants dropped out of the exercise intervention, resulting in an overall attrition rate of 11% (4% less than anticipated). Reasons for withdrawing were health-related (n = 3), noncommittal (including time conflict) (n = 4), and lost to follow-up (n = 1) (Fig. 1). On the study outcome data ascertainment, 65 participants (94%) provided both baseline data and end-point data at week 16. We lost 4 participants (6%) at the 16-week follow-up assessment due to change in health conditions (n = 2), being unwilling to complete (n = 1), and lost to follow-up (n = 1).

### Intervention Acceptability

For the cognitively enhanced online Tai Ji Quan group, descriptive statistics showed that 77% of the participants “strongly agreed” (“5”) and an additional 23% “agreed” (“4”) that the exercise program was safe to practice. A total of 55% “strongly agreed” and 45% “agreed” that the exercises were challenging but enjoyable. A total of 59% “strongly agreed” and 36% “agreed” that the intensity of the exercise program was appropriate and manageable. A total of 64% rated the program as “extremely helpful” for brain health, with 27% finding it “very helpful” and 9% finding it “moderately helpful.” Finally, on overall satisfaction, 82% were “extremely satisfied” and 18% were “somewhat satisfied” with the program (Table 2).

**Table 2:** Intervention acceptability rated by participants in the cognitively enhanced online Tai Ji Quan intervention (N = 22^a^)

Item/Rating Scale	Strongly agree	Agree	Neutral	Disagree	Strongly disagree
1. I found the exercise program safe to practice (n, %)	17 (77)	5 (23)	0	0	0
2. I found the exercises challenging but enjoyable (n, %)	12 (55)	10 (45)	0	0	0
3. I found the intensity of the exercise program appropriate and manageable (n, %)	13 (59)	8 (36)	1 (5)	0	0
	Extremely helpful	Very helpful	Moderately helpful	Little helpful	Not at all helpful
4. How helpful did you find the exercise program to improve your brain health overall? (n, %)	14 (64)	6 (27)	2 (9)	0	0
	Extremely satisfied	Somewhat satisfied	Neither satisfied nor dissatisfied	Somewhat dissatisfied	Extremely dissatisfied
5. Overall, how satisfied were you with the exercise program? (n, %)	18 (82)	4 (18)	0	0	0

^a^One participant was unable to complete the survey due to health reasons

### Adverse Events

No serious adverse events were documented over the 16-week study period (Table 3). A total of five moderate events (related to medical surgeries or conditions) were reported, none of which was intervention related. Of the six mild events reported, four were either possibly or definitely intervention related. For example, 1 participant in the cognitively enhanced Tai Ji Quan group reported a hernia pain caused by the exercise and 3 participants complained about muscular discomfort or pain. Of the 11 participants who reported any adverse events, all but 3 successfully completed the assigned intervention and outcome assessment.

**Table 3:** Number of adverse events reported during the 16-week study period by intervention group (N = 69)

	Intervention Group								
Nature of event*	Cognitively Enhanced Tai Ji Quan								
	Tai Ji Quan			Standard Tai Ji Quan			Stretching		
	Relationship to Intervention								
	Unrelated	Possible	Definite	Unrelated	Possible	Definite	Unrelated	Possible	Definite
**Serious**									
Number of events	0	0	0	0	0	0	0	0	0
**Moderate**									
Number of events	1	0	0	1	0	0	3	0	0
**Mild**									
Number of events	0	1	1	1	1	1	0	1	0

^*^Definition:

**Mild**: events that required no medical treatment and were non-life threatening (e.g., lower back pain, ankle/muscle soreness or pain, a fall without needing medical attention).

**Moderate**: events that required medical treatment but were not immediate life-threatening conditions (e.g., hand surgery or a medical procedure).

**Serious**: death or life-threatening events that required medical treatment, including hospitalization (e.g., open heart surgery or a major medical surgery that required hospital admission), or that resulted in significant disability/incapacity.

### Online Data Ascertainment

We received no complaints about online assessment from participants. In addition, participants who completed their online baseline assessment (N = 69) and 16-week follow-up assessment (n = 65) encountered no major issues with respect to device/Zoom use, Internet connection, movement space for the dual-task walking measures, or safety concerns. The average time to complete the online assessment of the cognitive and gait performance measures was approximately 45 minutes. Ninety-one percent of the study participants submitted the study survey online. All study informants completed their CDR evaluation for their respective participant.

We found no major break in our blinding assessment protocol during the study. One assessor was exposed, by accident, to a participant’s group assignment during scheduling for a follow-up assessment and was immediately replaced by another blinded assessor.

### Test-Retest Reliability

Reliability estimates for the cognitive measures are presented in Table 4. ICCs from the 2-week test-retest analyses were quite high, ranging from 0.87 (MoCA) to 0.92 (Verbal Fluency).

**Table 4:** Intraclass correlation coefficients from test-retest (at a 2-week interval) of cognitive measures

Cognitive measures	
	ICC
Montreal Cognitive Assessment	0.87
Trail-Making B	0.91
Digit Span - forward	0.90
Digit Span - backward	0.89
Verbal Fluency	0.92

*ICC* Intraclass correlation coefficient

### Change in Cognitive and Dual-Task Outcomes

Table 5 presents descriptive statistics related to the cognitive and dual-task cost measures at baseline and 16 weeks, and change scores from baseline. At week 16, a change from baseline, judging from the CI estimates, could be observed for all outcome measures for the cognitively enhanced Tai Ji Quan group. Similarly, with the exception of dual-task cost, a change was also noted on the outcome measures for the standard Tai Ji Quan training group. No change was evident on all the estimates for the stretching exercise group.

**Table 5:** Descriptive statistics (means and standard deviations) on cognitive and dual-task cost measures at baseline and 16 weeks, and mean change scores (with 95% confidence intervals) from baseline

Cognitive and dual-task cost measure	Cognitively	Standard	
	Enhanced Tai Ji Quan (n = 23)	Tai Ji Quan (n = 22)	Stretching (n = 24)
Montreal Cognitive Assessment (points), mean (SD)			
Baseline 16 weeks Change from baseline (16 weeks – baseline), (95% CI)	25.04±2.87 27.39±1.37 2.35 (1.18 to 3.51)	25.09±2.43 26.82±1.84 1.73 (0.67 to 2.79)	25.13±2.19 25.54±1.89 0.42 (-0.29 to 1.12)
Dual-task cost (%), mean (SD)			
Baseline 16 weeks Change from baseline (16 weeks – baseline) (95% CI)	38.38±23.06 13.54±15.96 -24.83 (-34.79 to -14.87)	39.89±28.64 28.46±19.13 -11.43 (-27.40 to 4.54)	38.58±22.19 47.69±34.53 9.11 (-2.69 to 20.92)
Trail Making Test B (seconds), mean (SD)			
Baseline	95.36±8.08	95.38±11.68	95.49±11.35
16 weeks	69.81±13.91	84.67±14.67	93.39±11.59
Change from baseline (16 weeks – baseline) (95% CI)	-25.55 (-32.94 to -18.17)	-10.70 (-14.70 to -6.69)	-2.11 (-6.74 to 2.53)
Forward Digit Span (points), mean (SD)			
Baseline	10.57±1.41	10.64±1.05	10.63±1.25
16 weeks	12.48±1.73	12.32±1.78	11.25±2.05
Change from baseline (16 weeks - bassline) (95% CI)	1.91 (1.26 to 2.56)	1.62 (1.08 to 2.28)	0.63 (-0.02 to 1.27)
Backward Digit Span (points), mean (SD)			
Baseline	8.26±0.86	8.27±1.42	8.33±1.13
16 weeks	9.87±1.25	9.32±1.56	8.13±1.98
Change from baseline (16 weeks - bassline) (95% CI)	1.61 (1.09 to 2.13)	1.05 (0.51 to 1.58)	-0.21 (-1.11 to 0.69)
Verbal Fluency (number of animals named), mean (SD)			
Baseline	13.26±1.66	13.36±0.95	13.71±1.39
16 weeks	17.22±2.59	15.59±2.30	14.13±1.36
Change from baseline (16 weeks - bassline) (95% CI)	3.96 (4.95 to 2.97)	2.23 (1.09 to 3.36)	0.42 (-0.24 to 1.08)

*SD* Standard deviation

## Discussion

In this 3-arm feasibility trial, we examined the feasibility, acceptability, and safety of implementing a newly developed cognitively enhanced online Tai Ji Quan intervention, along with a standard Tai Ji Quan training and stretching exercise, for older adults with MCI. Feasibility was demonstrated by the successful online program implementation with acceptable fidelity, intervention compliance, and study retention. The newly developed intervention was perceived to be acceptable and satisfactory by the participants. Overall, participants in the study completed the assigned exercise intervention, with no major intervention-related serious or moderate events observed across all intervention groups.

Recruitment of the study population was shown to be feasible for such an online (virtual) intervention. We were able to successfully carry out our online recruitment procedures (prescreening per the study eligibility criteria, study consent) with an approximately 2-to-1 screening-to-enrollment ratio, or a recruitment rate of 55%. Of the different recruitment promotion methods used, mass mailing and word of mouth were the most successful methods of reaching out to the target population [27]. The recruitment started in the state of Oregon and extended to other states, reflecting general interest in both the use of and receiving telehealth services [41, 42]. Even with different time zones, our online class was able to accommodate older adults in other states, depending on individual schedules. This was encouraging because it potentially helps the program reach a broader population, including those who may have been left behind [43] and those who are underserved and living in remote areas or rural settings. The intervention attrition (dropout) rate was 11%, and we were able to retain 94% of the study participants. Reasons for dropping out of the study were similar to previous in-person interventions [27, 28, 31, 32], and none of the reasons were related to the online class set-up or use of Zoom technology.

To accommodate online data collection, modifications were made in some cognitive measures, which were developed based on in-person protocols. Even with alterations in measurement set-up and testing protocols, we found no safety or technical issues during online assessment, which was conducted via videoconferencing at the participants’ homes, thus showing the feasibility of collecting cognitive and mobility outcome data remotely and with good test-retest reliability. These results, along with those we demonstrated previously [27], provide support for ascertaining online data when, under extenuating circumstances, in-person data collection approaches are not feasible, such as with COVID-19 or for those living in remote areas. Finally, changes (in the sense of improvement) from baseline in the measured cognitive and dual-task outcomes were observed consistently for the cognitively enhanced group whereas the standard Tai Ji Quan training group improved in all cognitive measures but dual-task cost. No change in the outcome measures was observed for the stretching exercise group.

While the cognitive health benefits of Tai Ji Quan for cognitively intact and impaired older adults have been documented [16–18], to our knowledge this is one of the few studies that have considered Tai Ji Quan training with explicit integration of cognitive-motor dual-task performance and practice. Previously, we have shown the feasibility of an augmented dual-task Tai Ji Quan intervention for balance training and falls prevention for older adults with MCI [27]. In this feasibility study, we enhanced, and therefore extended, our prior work by synergistically combining the physical and cognitive elements underlying the goal-directed Tai Ji Quan movements to boost cognition in older adults with MCI. Our approach, which aligns with contemporary training strategies [44–46], extends current Tai Ji Quan training approaches by involving concurrent, interactive physical-cognitive exercises aimed at stimulating and challenging multiple cognitive domains. Unique features of our new intervention include (a) Tai Ji Quan practice intertwined with cognitive exercises, (b) active engagement of and interaction with participants by the instructor during online class sessions, and (c) a manual-driven protocol for online instructional teaching.

### Strengths and Limitations

A notable strength of this study is the rigorous 3-arm design and methodologies that involve both assessing trial process feasibility and exploring trial outcome measures. Other strengths include successful recruitment of the target population, implementation of online exercise intervention classes, and satisfactory intervention compliance and retention rates.

The feasibility study also has some notable limitations. First, no sample size calculations were performed due to the pilot nature of this study. Therefore, the results on the cognitive and dual-task outcomes are preliminary and serve as a trend in outcome measures for future trials. Similarly, our study population can only be confined to the eligibility criteria applied in this study. Second, our study design does not include a cognitive training arm. Therefore, we may not know whether multitasking cognitive training alone or Tai Ji Quan with multitasking training works equally in terms of potential efficacy of our reported cognitive outcomes. However, the inclusion of the standard Tai Ji Quan training in the study helps discriminate task-specificity in cognition and control for placebo effects, and thus allows a rigorous evaluation of the incremental value of adding a cognitive training component to our enhanced Tai Ji Quan training approach. Third, due to limitations associated with our online recruitment and assessment protocols, we were unable to include additional MCI screening measures, such as executive function, attention, language, or visuospatial skills, which would be more ideal in capturing the outcomes for the target population [47]. The same limitation exists in our cognitive outcome measures, which are to be used in our future efficacy trial. Inclusion of additional domain-specific outcome measures such as visuospatial, cognitive inhabitation, and memory-related functions would provide a better representation of cognition that may have been benefited from our intervention.

## Conclusions

In this 3-arm intervention feasibility trial, we demonstrated the feasibility, acceptability, and safety of a newly developed cognitively enhanced Tai Ji Quan intervention, delivered to home settings via online videoconferencing, for community-dwelling older adults with MCI. Additional feasibility data included collecting outcome data remotely and trends in cognitive and dual-task outcomes resulting from a 16-week intervention. We conclude that the outcomes from this feasibility study form the basis for a full-scale randomized online intervention trial that examines the effectiveness of our cognitively enhanced online Tai Ji Quan intervention for older adults with MCI.

## Data Availability

The de-identified data that support the findings of this study are available from the corresponding author upon reasonable request. Restrictions may apply to preserve participant confidentiality.

## Abbreviations

CDR: Clinical Dementia Rating
COVID-19: Coronavirus disease 2019
ICC: Intraclass correlation coefficient
MCI: Mild cognitive impairment
MMSE: Mini-Mental State Evaluation
MoCA: Montreal Cognitive Assessment
TUG: Timed Up and Go
TMT-B: Trail Making Test B
